# Pregnancy and COVID-19 Pandemic Perception in Malaysia: A Cross-Sectional Study

**DOI:** 10.3390/ijerph18115762

**Published:** 2021-05-27

**Authors:** Sharifah Aminah Syed Anwar Aly, Rahana Abdul Rahman, Shalisah Sharip, Shamsul Azhar Shah, Zaleha Abdullah Mahdy, Aida Kalok

**Affiliations:** 1 Department of Obstetrics and Gynaecology, Faculty of Medicine, National University of Malaysia, Universiti Kebangsaan Malaysia Medical Center, Cheras 56000, Malaysia; sharifahameena1010@gmail.com (S.A.S.A.A.); drrahana@ppukm.ukm.edu.my (R.A.R.); zaleha@ppukm.ukm.edu.my (Z.A.M.); 2 Department of Psychiatry, Faculty of Medicine, National University of Malaysia, Universiti Kebangsaan Malaysia Medical Center, Cheras 56000, Malaysia; shalisah@ppukm.ukm.edu.my; 3 Department of Community Health, Faculty of Medicine, National University of Malaysia, Universiti Kebangsaan Malaysia Medical Center, Cheras 56000, Malaysia; drsham@ppukm.ukm.edu.my

**Keywords:** COVID-19, knowledge, perception, practice, pregnancy

## Abstract

Pregnant women are susceptible to COVID-19 complications due to gestation-related physiological changes. We aimed to evaluate the level of maternal knowledge, perception, and practice during the pandemic. A cross-sectional study was conducted during the Malaysian Movement Control Order (MCO) between April and June 2020. A self-administered electronic questionnaire that included the knowledge and practice domains was distributed. A newly designed set of questions was used to evaluate (1) women’s perception of MCO and (2) maternal experience, which was subdivided into clinical care provision and maternal anxiety. The survey response rate was 93% with the final number for analysis of 415. The majority of women (95%) demonstrated an adequate level of knowledge on COVID-19, whilst 99% had a good practice. We found that tertiary education (*p* < 0.001), employment status (*p* = 0.03), higher household income (*p* < 0.001), and multiple sources of information (*p* < 0.001) were independent predictors of adequate maternal knowledge on COVID-19. Women with adequate knowledge also reported a more positive perception of MCO (*p* < 0.001) and better obstetric care experience (*p* = 0.037), as did those of Malay ethnicity. Younger (*p* < 0.001) and nulliparous (*p* = 0.01) women demonstrated greater anxiety levels. The majority of our women reported good practice and adequate knowledge, which contributed to a positive perception of MCO and better maternal obstetric experience. First-time mothers may benefit from extra support and reassurance during the pandemic to alleviate maternal anxiety.

## 1. Introduction

The Coronavirus disease 2019 (COVID-19) pandemic was a result of a novel RNA virus that caused a spectrum of respiratory infections from the mild, self-limiting disease to severe progressive pneumonia and acute respiratory distress syndrome (ARDS) [1,2]. Symptoms of COVID-19 include fever, dry cough, sore throat, anosmia, and dyspnea. Viral transmission can occur via direct or indirect route; through close contact with an infected person or touching the contaminated surfaces, respectively [3,4]. There has been a much wider and rapid viral spread with COVID-19, resulting in a higher ratio of infected persons and death compared to severe acute respiratory syndrome (SARS) or Middle East respiratory syndrome (MERS) [2,5,6].

The World Health Organization (WHO) declared a global pandemic on 11 March 2020, and to date, over 150 million people have been infected worldwide with a death toll of 3.2 million [2,7]. Malaysia has currently recorded over four hundred thousand confirmed cases with a mortality rate of 0.37% [8]. Malaysian Government has implemented the Movement Control Order (MCO) which aims to break the chain of COVID-19 in the population. The MCO prohibits mass gathering, stops international travel, and closes all academic and business premises except providers of essential services, including food, health, telecommunications, and transportation [9,10].

Globally, elective medical and surgical procedures have been cancelled in order to ensure adequate hospital resources to respond to COVID-19 pandemic. Although most of them are described as “elective”, these interventions are important contributors to the patients’ health and overall quality of life [11]. The delivery of healthcare in Malaysia, including obstetric service, has also been affected during the MCO. Antenatal care providers were instructed to limit the number of appointments to minimize overcrowding. This resulted in delayed antenatal appointments which may put those with complicated pregnancies at risk. The public had to adapt to the new norm which includes wearing a face mask, social distancing, frequent hand washing, and avoiding mass gatherings [12]. The Malaysian government have been using multiple platforms to convey official information and updates on COVID-19 to the general population, which include daily press conferences, regular text messaging, and the use of social media [10].

Pregnant women are more susceptible to COVID-19 complications due to the gestation-related physiological and immunity changes. The Centre for Disease Control and Prevention (CDC) reported that pregnant women with COVID-19 infection were more likely to be hospitalized, admitted to intensive care units, and requiring ventilator support [13]. Pregnant women’s adherence to disease preventative measures is crucial to minimize their risk of contracting the disease. Adherence to good practice is very much influenced by knowledge and attitude towards the pandemic, as demonstrated by previous studies [14,15]. We aimed to evaluate the maternal knowledge and practice during the COVID-19 pandemic, as well as maternal perception of the MCO and the obstetric care received. We hypothesized that women’s education level affects their knowledge. We also hypothesized that mothers with adequate knowledge will report a more positive perception towards the MCO and the clinical care they received.

## 2. Materials and Methods

### 2.1. Study Design and Participants

This cross-sectional study was conducted from May 2020 till June 2020 among women who received obstetric care in a teaching hospital in Kuala Lumpur. This study was approved by the Universiti Kebangsaan Malaysia Medical Research and Ethics Committee (FF-2020-211). The inclusion criteria were Malaysian women aged above 18 years old and able to understand the Malay language. Women with abnormal fetuses and stillbirth were excluded. Eligible participants were approached in the antenatal clinic as well as the obstetric ward. Consenting women were invited to complete the electronic version of the questionnaire through Google Forms. Socio-demographics and clinical data were also recorded.

### 2.2. Instruments

The survey questionnaire was formed based on discussion with a team of experts which consisted of epidemiologist, psychiatrist, and psychiatrist. The form was designed in Malay, the country’s national language. The survey consists of three domains that assess women’s knowledge, perception, and practices during the COVID-19 pandemic.

#### 2.2.1. Knowledge

The knowledge questionnaire involved nine items which covered the signs and symptoms of COVID-19, methods of transmission, and disease prevention. The response choices were “True”, “False”, and “Unsure”. A correct answer was given 1 point, whilst others scored 0 point. The total knowledge score ranged from 0 to 9. Participants’ overall knowledge was categorized into adequate if the score was more than 50% (5–9 points), and inadequate if the score was less than 50% (0–4 points).

#### 2.2.2. Perception towards the MCO and Maternal Experience during the COVID-19 Pandemic

The items in the perception domain were designed to evaluate the maternal perception of the MCO and obstetric care received during the pandemic. Participants were asked to respond to each statement using a scale: 1 (strongly disagree) to 7 (strongly agree).

Four items were used to assess the perception towards the MCO which are as follows:

1. The Movement Control Order is necessary.
2. Information from the authority is accurate and transparent.
3. I am willing to prolong the Movement Control Order if necessary.
4. My family and I have received enough support during the MCO.

The maternal experience on obstetric care received during the pandemic was evaluated through seven items as follows:

1. My antenatal appointments are not affected.
2. The staff (nurses/doctors) at the government clinic (or private general practice) were supportive and helpful.
3. The staff (nurses/doctors) at the hospital were supportive and helpful.
4. I feel anxious when my husband is not allowed to accompany me during my antenatal clinic appointment.
5. I have received clear advice on where to deliver or give birth.
6. I am worried about having to give birth during the Movement Control Order.
7. I feel anxious when my husband is not allowed to accompany me during childbirth.

The total score was calculated separately for each subscale, i.e., perception of MCO and maternal experience. A higher score would indicate a more positive perception or experience.

#### 2.2.3. Practices during COVID-19 Pandemic

Maternal practices were evaluated through five items: the frequency of using handwashing and hand sanitizer, wearing facemask, gloves, and frequency of going out. Good practices were given 2 points whilst poor practices scored 0 points. The total score of more than 50% (6–10) was considered as good practice.

### 2.3. Face Validation

Face validation was performed on 40 women who were not included in the final analysis. All women reported that the questionnaire was easy to understand and use.

### 2.4. Statistical Analysis

The sample size was determined using the Epi Info 7 software. Assuming that the probability of having adequate knowledge and a positive attitude was 50.0% [16], at 95% confidence interval, the limit of precision 5%, with a design effect of 1.0, the calculated sample size was 384 participants.

The study data were analyzed using the Statistical Package of Social Sciences (SPSS) Version 24.0 (IBM Corp., Armonk, NY, USA). Data were presented as mean (standard deviation, SD) or number, n (percentage, %) for continuous and categorical data, respectively.

The reliability of the perception questionnaire was assessed using Cronbach’s alpha. Cronbach’s alpha > 0.7 was regarded as satisfactory. All of the items in the perception domain underwent exploratory factor analysis via orthogonal (varimax) rotation to confirm the number of factors. The Bartlett’s test of sphericity and the Kaiser–Meyer–Olkin (KMO) test were used to evaluate sampling adequacy, whilst the Kaiser rule (Eigenvalue > 1.0) was applied to determine the number of dimensions to extract. The scores for the perception domain were inspected for normality using the Kolmogorov–Smirnov test.

Univariate analysis was performed to determine the factors associated with adequate knowledge and good practice. The statistically significant factors were then analyzed using the multiple variable logistic regression to produce the adjusted odd ratios and the corresponding 95% confidence interval. For the perception domain, the mean scores for maternal perception and experience subscales were compared among women with different socio-demographic and clinical factors. A *p*-value less than 0.05 was considered statistically significant.

## 3. Results

A total of four-hundred and fifty women were approached and 420 completed the questionnaire, resulting in a response rate of 93%. Five participants were excluded due to incomplete entries, making our final number for analysis 415. Table 1 demonstrates the demographics data of our study cohort. The mean age of our respondents was 32.4 (4.5). The majority of the participants (85.1%) were Malays. Around three-quarters of the participants received tertiary level of education and were employed. Almost 60% were antenatal mothers, and just over a third of our cohort had a medical or pregnancy-related disorder.

The majority of our women stated their sources of knowledge were television (80.2%), followed by the internet (76.1%) and social media such as Twitter, Instagram, and Facebook (73.5%). More than half of the participants (68%) received information on COVID-19 from social messaging such as WhatsApp, Telegram, and public text messaging, while 17.3% read newspapers.

The results of the knowledge survey are shown in Table 2. The mean knowledge score for our cohort was 7.67 (1.5). A total of 95% of women demonstrated adequate knowledge on COVID-19, with over four-fifths of our respondents answered all questions correctly.

We found that tertiary education, employment status, household income (>RM 5000), and the higher number of information sources were significantly associated with adequate maternal knowledge on COVID-19. These factors remained significant following adjustment for age, ethnicity, and parity in the multivariable logistic regression analyses (Table 1).

Table 3 demonstrates the participants’ perception towards the Movement Control Order and antenatal care during the COVID-19 pandemic. More than four-fifths of the participants believed that the Movement Control Order (MCO) is necessary (81.7%) and are willing to prolong it if necessary (85.0%). Three hundred and fifty-four participants (85.3%) agree that information from authority is accurate and transparent. Only less than one-fifth of the participants felt that they did not receive enough support during the MCO. Table 4 shows the maternal experience during the pandemic. Positive perception towards antenatal care was high among our cohort study, with almost 90% of them feeling that primary and tertiary centers were supportive and helpful during the pandemic.

The Cronbach’s alpha for maternal perception of the MCO and maternal experience were 0.91 and 0.81, respectively, indicative of good internal consistency. The Kaiser–Meyer–Olkin (KMO) was 0.825 (MCO perception) and 0.752 (maternal experience), and Bartlett’s test of sphericity reached statistical significance with *p* < 0.001 for both subscales, supporting the sample factorability. Exploratory factor analysis on the four items of MCO perception confirmed single factor loading. The maternal experience questionnaire demonstrated two-factor loading. Because of this, we decided to analyze the scores under two subdomains, i.e., obstetric care (items 1–3 and 5) and maternal anxiety (items 4, 6, and 7).

Table 5 compares the mean scores for MCO perception and maternal experience during the pandemic (with obstetric care and maternal anxiety subdomains) between different groups. Higher scores are indicative of more positive perception, better experience, and greater anxiety. The Mann–Whitney U test was used to analyze our data as the scores were not normally distributed.

We found that Malay women (*p* < 0.001) and mothers with a medical condition (*p* = 0.003) reported more positive perceptions towards the MCO. Respondents with adequate knowledge on COVID-19 (*p* < 0.001), as well as those who received information from multiple sources (more than three) (*p* = 0.049), had greater MCO perception scores. Women with adequate knowledge also reported better obstetric care (*p* = 0.037). Malay women reported better experience when receiving clinical care during the pandemic than the non-Malays (*p* = 0.002). Younger (*p* < 0.001) and nulliparous women (*p* = 0.010) demonstrated a higher level of anxiety, whilst those in the critical sector employment (*p* = 0.041) and with the medical condition (*p* = 0.028) reported lower scores in the anxiety subscale.

Table 6 portrays the behavioral changes during the COVID-19 outbreak. The prevalence of good practice among our study cohort was 99%. The majority of the respondents practiced good hand hygiene and wore a mask every time leaving their house. More than half of the respondents (52.0%) practiced preventive measures by staying at home and only leaving home when necessary.

## 4. Discussion

We presented the first Malaysian study which explored the knowledge, perception, and practice among pregnant women during the COVID-19 pandemic. In our well-educated urban population, we found that 95% of respondents demonstrated adequate knowledge about COVID-19. The majority of women had a favorable perception towards government efforts in combating the COVID-19 outbreak and believed that they received enough support from health professionals despite the pandemic.

Various factors contributed to the high level of knowledge among our cohort. Previous studies on COVID-19 knowledge have shown that female respondents generally displayed greater scores in comparison to their male counterparts [15,17,18,19]. Higher education level is a positive predictor of adequate knowledge on COVID-19, as shown by various population studies [17,20,21,22]. The overall correct rate for the knowledge questionnaire among our cohort was 85.2%, which was in keeping with other local studies [18,23]. This was unsurprising considering that three-quarters of our respondents held a tertiary qualification. Zhong et al. demonstrated a correct rate of 90% on the knowledge questionnaire among their predominantly women, well-educated population in China [17].

Our study also found that maternal employment and greater household income were independently associated with adequate COVID-19 knowledge. Higher socioeconomic status was linked to good COVID-19 knowledge and practice in studies from China, Saudi Arabia, and Egypt [15,17,22]. Similarly, a study among pregnant women in Iran demonstrated a positive correlation between socioeconomic status and adequate knowledge scores, with women in the urban areas reporting higher scores than their rural counterparts [24]. Our urban cohort was more likely to have unlimited access to the internet and various online information resources, in comparison to rural, less-educated women with lower household income. Multiple sources of information also contributed to greater knowledge among our women. The use of the internet had exponentially increased with the onset of COVID-19. The MCO restriction to various daily activities resulted in increased demand for home-based entertainment, video conference, and online communication [25]. More people began to share and express their thoughts, emotions, and ideas online due to decreased options of more personal face-to-face interaction. This ultimately led to the growing number of social networking service users, with an estimated 26.1 million Malaysian users by 2023 [25]. As of January 2021, about 86 percent of the Malaysian population were active social media users, with Facebook being the most widely used platform [26]. Unsurprisingly, almost three-quarters of our respondents were Facebook users. The Malaysian government had recognized the powerful influence of social media on the masses and began to disseminate public health messages through official social network accounts on Facebook and Instagram, alongside traditional channels such as television, radio, and newspaper. Malaysians also received COVID-19 updates through mobile phone text messages.

Clear and transparent information from the authority is essential to dispel any myths or misinformation regarding the pandemic, as several studies demonstrated that uncertainties and misconceptions about COVID-19 were widespread among the general public [20,27]. The government’s prompt response to safeguard the nation against COVID-19 by implementing stringent control and precautionary measures such as lockdown and travel ban had encouraged a positive attitude and high confidence in the pandemic control among the public. Al-Hanawi et al. found that 97% of the Saudi population were convinced that the government will control the COVID-19 outbreak. A recent study in China also found that the majority of respondents were confident that the country can win the battle against COVID-19 [17]. A study performed by Azlan et al. showed that Malaysians had positive attitudes towards the successful control of COVID-19 by the government (83.1%) and the Movement Control Order (89.9%) [18]. These findings were similar to our study, where over four-fifths of our women felt that the MCO was necessary and 85% agreed that it should be prolonged if necessary. Adequate knowledge and multiple sources of information are amongst independent predictors of positive perception towards MCO among our cohort. A review of studies conducted during the COVID-19 pandemic demonstrated that knowledge directly influenced attitudes [28]. Our finding is similar to that of Zhong et al., who found that good COVID-19 knowledge was associated with an optimistic attitude.

Adequate maternal knowledge was also associated with positive obstetric experience among our respondents. Health service delivery, including obstetrics, was inevitably disrupted worldwide by the COVID-19 outbreak. Compromised quality of care, unfortunately, led to an increase in maternal and neonatal mortality, especially in a low-resource setting [29,30,31]. Our tertiary center was one of the designated COVID-19 hybrid hospitals in which routine health care service was delivered alongside treatment for COVID-19 patients. More than two-thirds of our women reported that their antenatal appointments were not affected by the pandemic and around 90% felt that they received good support from the health care providers. Pregnant women were observed to trust the health professionals (92.4%) and reported an increased level of respect towards them (82.5%) during the pandemic [32]. Maternal cooperation during the worldwide pandemic is valuable in reducing COVID-19 transmission risk to both mother and baby. Malay women in our cohort reported a more favorable perception towards the MCO and obstetric care. This may be explained by the underlying good knowledge of COVID-19 and high regard towards health professionals and authority. A survey conducted in Singapore during the start of the pandemic found that pregnant Malay women were more likely to practice safe distancing and frequent hand sanitizing compared to the Chinese [33].

The prevalence of anxiety among pregnant women during the COVID-19 outbreak was reported between 20.8% and 43.9% [34,35,36]. Threat to pregnancy and low perceived control were two significant contributors to pregnancy-related anxiety. Risk of COVID-19 infection to the fetus, uncertainties regarding delivery, fear of income loss, and family conflict were identified as important stressors to pregnant women during the pandemic [37,38]. Our questionnaire explored maternal anxiety concerning giving birth during the MCO and the absence of a partner during clinic appointments as well as delivery. Adhikari et al. found that 73.4% of their cohort worried about childbirth plan and place [38], a finding similar to ours. We found that younger women (age 35 and below) and nulliparous demonstrated a greater level of anxiety. The lack of a partner’s support during childbirth is understandably causing more distress among mothers who were undergoing labor for the first time. Interestingly, women who worked in the critical sector and had underlying medical conditions displayed lesser maternal anxiety. Lee et al. found that pregnant women who worked as front-liners sanitized their hands at higher frequency whilst women with high-risk pregnancies displayed more tendency to stay at home. These findings reflect good awareness and practice against COVID-19 among them. It was unsurprising that these women found measures to reduce overcrowding (in clinic and labor room) appropriate, resulting in a lower score of anxiety.

### Study Strength and Limitations

We believe that our study is the first to assess maternal knowledge, perception, and practice in Malaysia at the early stage of the COVID-19 outbreak. Our newly designed questionnaire on the perception of the Movement Control Order (MCO) and maternal experience demonstrates good internal consistency and construct validity. The electronic version of our questionnaire made data collection quicker and more efficient, allowing similar research to be replicated in a different cohort easily. Our reasonably large sample size was also adequate to meet the statistical requirement.

Our research findings were similar to those of other studies worldwide. As the majority of our women were well-educated and employed with a middle-class income, this study’s results are only generalized to urban pregnant women of high economic status. Further research needs to be conducted among women from rural areas, as well as those with lesser income and limited access to the internet or online resources.

Our study was also limited by its cross-sectional nature. This survey was conducted during the beginning of the country’s partial lockdown, where there was a high level of anxiety among the population. The ongoing pandemic has resulted in the continuation of the nationwide MCO. A longitudinal study on the effect of prolonged movement restriction on maternal mental health is crucial so that appropriate support can be delivered.

## 5. Conclusions

In summary, a high level of knowledge and good practice against COVID-19 infection among urban pregnant women in Malaysia was most likely due to their high economic status. Women with adequate knowledge reported favorable perception towards the Movement Control Order and experienced better obstetric care. Younger and first-time mothers are more susceptible to anxiety and would benefit from extra support during the ongoing pandemic.

## Figures and Tables

**Table 1:** Demographic characteristics and respective adjusted odd ratios for adequate knowledge on COVID-19.

Characteristics	*n* (%)	AOR *	(95% CI)	*p*-Value
Age				
≤35 years old	296 (71.3)	1.00	(0.47, 2.83)	0.76
>35 years old	119 (28.7)	1.15		
Ethnicity				
Malay	353 (85.1)	2.50	(0.93, 6.78)	0.07
Non-Malays	62 (14.9)	1.00		
Parity				
Nulliparous	68 (16.4)	2.04	(0.46, 9.12)	0.35
Multiparous	347 (83.6)	1.00		
Level of education				
Primary–Secondary	96 (23.1)	1.00	(2.92, 20.13)	<0.001
Tertiary	319 (76.9)	7.66		
Employment status				
Employed	321 (77.3)	3.98	(1.61, 9.85)	0.03
Non-employed	94 (22.7)	1.00		
Essential sector employment				
Yes	157 (37.8)	1.71	(0.60, 4.92)	0.32
No	258 (62.2)	1.00		
Monthly household income				
<RM 5000	203 (48.9)	1.00	(3.32, 191.3)	<0.001
≥RM 5000	212 (51.1)	25.19		
Pregnancy period				
Antenatal	240 (57.8)	2.27	(0.67, 5.93)	0.09
Postnatal	175 (42.2)	1.00		
Mode of delivery				
Vaginal delivery	107 (61.1)	1.00	(0.59, 4.03)	0.59
Operative Delivery	68 (38.9)	1.54		
Medical Condition (pre-existing and pregnancy-related)				
No	260 (62.6)	1.00	(0.60, 4.33)	0.34
Yes	155 (37.4)	1.61		
Source of information				
1–3 sources	220 (53.0)	1.00	(1.87, 38.02)	<0.001
More than 3 sources	195 (47.0)	8.66		

Abbreviations: AOR, adjusted odds ratio; CI, confidence interval; * AOR adjusted to age, ethnicity, and parity using multivariable logistic regression

**Table 2:** Assessment of maternal knowledge on COVID-19.

Items	Correct Answer *n* (%)
Symptoms of COVID-19	
Fever with a body temperature of more than 38 °C.	388 (93.5)
Cough and sore throat.	386 (93.0)
Difficulty of breathing.	367 (88.4)
Mode of transmission of COVID-19 virus	
Coughing and sneezing by an infected person.	385 (92.8)
Direct physical contact with an infected person.	392 (94.5)
Touching or coming into contact with surfaces or objects contaminated with the virus.	337 (81.2)
The minimal time for handwashing recommended by the Ministry of Health is 20 s.	212 (51.1)
The social distance recommended by the Ministry of Health is one meter.	390 (94.0)
There is no treatment and vaccination for COVID-19.	332 (80.0)

**Table 3:** General perceptions on the Movement Control Order (MCO) during the COVID-19 pandemic.

Items		Agree	Neutral	Disagree
		*n* (%)	*n* (%)	*n* (%)
1	The MCO is necessary.	339 (81.7)	20 (4.8)	56 (13.5)
2	Information from the authority is accurate and transparent.	354 (85.3)	19 (4.6)	42 (10.1)
3	I am willing to prolong the MCO if necessary.	353 (85.0)	19 (4.6)	43 (10.4)
4	My family and I have received enough support during the MCO.	313 (75.4)	32 (7.7)	70 (16.9)

**Table 4:** Maternal experience during COVID-19 pandemic.

Items		Agree	Neutral	Disagree
		*n* (%)	*n* (%)	*n* (%)
1	My antenatal appointments are not affected.	295 (71.1)	27 (6.5)	93 (22.4)
2	The staff (nurses/doctors) at the government’s clinic (or private general practice) were very supportive and helpful.	371 (89.4)	10 (2.4)	34 (8.2)
3	The staff (nurses/doctors) at the hospital were very supportive and helpful.	372 (89.6)	12 (2.9)	31 (7.5)
4	I feel anxious when my husband is not allowed to accompany me during the antenatal clinic appointment.	224 (54.0)	47 (11.3)	144 (34.7)
5	I have received clear advice on where to deliver/give birth.	267 (64.4)	50 (12.0)	98 (23.6)
6	I am worried about having to give birth during the MCO.	292 (70.4)	37 (8.9)	86 (20.7)
7	I feel anxious when my husband cannot accompany me during childbirth.	303 (73.0)	30 (7.2)	82 (19.8)

**Table 5:** Mean scores comparison between different socio-demographic and clinical factors.

Groups	Perception of MCO	Maternal Experience	
		Obstetric Care	Anxiety
Overall score, mean (SD)	24.05 (5.48)	22.78 (5.44)	15.15 (5.28)
Age			
≤35	24.17	23.02	15.78
>35	23.85	22.39	14.11
*p*-value	0.373	0.189	<0.001
Ethnicity			
Malay	24.53	23.16	15.14
Non-Malay	21.31	20.61	15.19
*p*-value	<0.001	0.002	0.983
Education			
Tertiary	24.16	22.71	15.04
Non-tertiary	23.69	23.02	15.52
*p*-value	0.837	0.098	0.170
Critical sector employment			
Yes	24.43	22.61	14.55
No	23.82	22.88	15.51
*p*-value	0.664	0.442	0.041
Parity			
Nulliparity	24.25	23.09	16.72
Multiparity	24.01	22.72	14.84
*p*-value	0.704	0.768	0.010
Medical condition			
Yes	24.59	23.05	14.39
No	23.72	22.61	15.61
*p*-value	0.003	0.364	0.028
Source of information			
1–3	23.41	22.63	15.05
more than 3	24.76	22.95	15.25
*p*-value	0.049	0.805	0.636
Knowledge adequacy			
Yes	24.38	22.96	15.29
No	17.90	19.38	12.43
*p*-value	<0.001	0.037	0.059

**Table 6:** Practices during the COVID-19 outbreak.

Items		Less *n* (%)	No Change *n* (%)	More *n* (%)
Frequency of handwashing		2 (0.5)	3 (0.7)	410 (98.8)
Frequency using hand sanitizer		8 (1.9)	8 (1.9)	399 (96.1)
Frequency wearing facemask		8 (1.9)	7 (1.7)	400 (96.4)
Frequency wearing gloves		124 (29.9)	120 (28.9)	171 (41.2)
	**Only When Necessary** ***n* (%)**	**1–2 Times a Week** ***n* (%)**	**4 to 5 Times a Week** ***n* (%)**	**Daily** ***n* (%)**
Frequency going out	216 (52.0)	127 (30.6)	46 (11.1)	26 (6.3)
